# Convergence and determinants of health expenditures in OECD countries

**DOI:** 10.1186/s13561-017-0164-4

**Published:** 2017-08-17

**Authors:** Son Hong Nghiem, Luke Brian Connelly

**Affiliations:** 1 The Australian Research Centre for Health Services Innovation, Institute of Health and Biomedical Innovation, School of Public Health and Social Work, Queensland University of Technology, Brisbane, QLD 4059 Kelvin Grove Australia; 2 Centre for the Business and Economics of Health, Faculty of Health and Behavioural Sciences, The University of Queensland, Brisbane, QLD 4072 Australia

**Keywords:** Health expenditure, Convergence, OECD countries

## Abstract

This study examines the trend and determinants of health expenditures in OECD countries over the 1975-2004 period. Based on recent developments in the economic growth literature we propose and test the hypothesis that health care expenditures in countries of similar economic development level may converge. We hypothesise that the main drivers for growth in health care costs include: aging population, technological progress and health insurance. The results reveal no evidence that health expenditures among OECD countries converge. Nevertheless, there is evidence of convergence among three sub-groups of countries. We found that the main driver of health expenditure is technological progress. Our results also suggest that health care is a (national) necessity, not a luxury good as some other studies in this field have found.

## Background

Rising real per capita incomes, technological innovation and ubiquitous insurance against medical treatment and the ageing of the population are generally considered to exert important influences on the growth of health expenditures. The causal inter-relationships between these factors, though, are complex. Despite common perceptions, the bulk of health expenditure growth is not due to population ageing *per se*[19, 49], but the growth in demand for new medical technologies (MTs) that improve and/or extend life as real per capita incomes grow [15, 20, 49, 71, 73]. Yet the foregoing statement—while true—is also deceptively simplistic. First, since shares of GDP not only reflect expenditures but income, it is true not only to say that national income drives health expenditures, but also that health expenditures drive national income growth. The link between health *per se*and growth and health and productivity has been explored by a number of authors (Narayan et al. [45] provide an overview; also see Pradhan [59]). Second, the distinction between age-related and technology-related sources of health expenditure growth is likely to be—at least in part—a false distinction. The demand for technological innovation in the health sector will increase not only with income, but also with needs, many of which are correlated with ageing. Finally, there is also some recent and somewhat contradictory evidence from the US [7] that the health sector suffers from Baumol’s [9] “cost disease”. Specifically, this empirical work suggests that the health sector is a “non-progressive” sector of the economy, being characterised as labour-intensive and relatively devoid of innovations that enhance labour productivity. The latter results are curious and probably have more to do with measurement problems than, as the authors suggest “…relatively constant productivity and stagnant technology…” (Bates & Santerre [7], p. 386). Yet, in addition to the obvious microeconomic and econometric issues that are still to be resolved in respect of the drivers of health expenditure, there are also some important macroeconomic dimensions of health expenditure growth that are yet to be explored. The purpose of this paper is to use recent developments in the economic growth literature to address the question of whether or not health expenditure growth across nations tends to converge over time. This question is of interest because if the rate of health expenditure growth for countries does converge over time, this effect may attenuate (or exacerbate) the rates of growth that might otherwise be estimated and predicted from microeconometric work on this topic. Clearly, if such a phenomenon were at work, it may have important implications for public policy and planning with respect to the health sector. Health expenditures account for a large proportion of the Gross Domestic Products (GDP) in each of the Organization for Economic Cooperation and Development (OECD) countries and have grown considerably over the past few decades. For instance, the median health expenditure in the OECD increased from 3.8% in 1960 to 7.9% in 1990 [2]. Our observations from OECD health data in the 1975–2004 period also reveal that the growth of health expenditure per capita consistently exceeds the growth of GDP per capita. In addition, health expenditure growth is faster in more affluent countries, and the health sector accounts for a greater proportion of GDP in those countries. For example, the proportion of health expenditures in GDP of the United States– the richest country in the OECD– in 1960 and 1998 was 5.2 and 14.0%, respectively. The level of total health expenditure per capita (measured in purchasing power parity) of the United States also consistently among the highest in the OECD for the period 1975–2004.

Common features of developed countries (e.g., aging population, technological advancement, high coverage of health insurance), all of which positively affect the cost of health care, lead us to form a hypothesis that health expenditure in countries of similar economic development level converge over time. To the best of our knowledge, only a limited number of previous studies, including Hitiris [36]; Barros [6]; Nixon [50]; Hitiris & Nixon [37]; Narayan [44]; Panopoulou & Pantelidis [53]; Lau et al. [41]; Pekkurnaz [55], have examined the convergence of health expenditure among OECD countries. However, the standard convergence tests in the economic growth literature (i.e., *β*− and *σ*−convergences) applied in Barros [6] and Nixon [50] assume that all countries follow the same growth path. The unit root test procedure applied by Narayan [44] is more flexible but it was focused on structural breaks at the level (rather than on the growth) of health expenditure and accommodates no more than two breaks.

This study contributes to the literature by examining the convergence of the growth in health expenditure in OECD countries using the dynamic growth model by Phillips & Sul [58]. The advantage of this approach is that it allows individual countries to follow distinctive growth paths. In addition, we examine the determinants of health expenditure growth using panel data methods, which confer several further econometric advantages over some of the previous work on this topic.

## A brief review of the literature

It is expected, based on the available literature, that technology will be the major determinant of health expenditure. Much of the existing literature on this topic, though, suffers from the use of econometric techniques—such as standard ordinary least-squares (OLS) regression—to analyse time-series data. This is now known to be problematic as, typically, health expenditure (HE) and GDP are cointegrated [27]. Modern time-series econometric techniques are able to overcome the possibly spurious results [76] that can be associated with regressing non-stationary cointegrated time-series. Given the statistical problems that beset the historical literature on this topic, it is interesting that research tends to confirm its long-standing and somewhat counter-intuitive result: technically (according to the standard economic definition), health care is a “luxury”. Specifically, spending in the health sector tends to rise at a faster rate than national income. Indeed the “income elasticity of HE” (i.e., the percentage change in HE for a percentage change in GDP) usually well exceeds unity at the national level for the Organization for Economic Cooperation and Development (OECD) countries (see e.g., [17]). The most recent contribution to the applied literature [73] for example, estimates that the income elasticity of HE in the US over a 40-year period was approximately 1.388 to 1.445. The most recent results for Australia were based on OECD data from 1960 to 1997 and produced a similar point estimate of 1.47 (Clemente et al. [17], Table 2, p.598).

There are considerable differences in the size of the health sector among OECD countries: in 2010, HE comprised 6.9% of GDP in Mexico and 17.4% of GDP in the USA [39]. On a per capita basis too, the US is an outlier, with the US spending in more per capita than the mean of other high-income countries [19]. Given these differences and the remarkable heterogeneity of insurance arrangements, practitioner remuneration arrangements, regulatory controls and so forth, it is also astounding that the rate of growth of HE per capita has long been shown to be similar across the developing world (see, e.g. [17, 21, 48, 49]). This has led economists to question whether or not the rates of health expenditure growth across countries might tend to converge over time [6, 36, 51]. The most recent study in this genre [44] examined the health expenditure growth of six countries (not including Australia) for the period 1960–2000. It found that there was evidence of the convergence of per capita health expenditures of the UK, Canada, Japan, Switzerland and Spain to that of the USA.

The trends and determinants of health expenditure in developed countries have been widely examined and revealed that main determinants of health expenditure growth include: income growth [1, 6, 14, 30, 49], the ageing of the population [31, 43, 68], technological progress [3, 15, 16, 52, 75], and the widespread availability of health insurance [13, 22, 24, 29, 57, 70]. Newhouse [48] is the most cited study examined the relationship between income and health expenditure. In particular, Newhouse [48] showed that GDP per capita explained more than 90 percent of the variations in health expenditure per capita in OECD countries, and hence health services belong to the group of “luxury” goods (i.e., income elasticity is greater than one). However, results of other studies were mixed: the income elasticity of health expenditure was found to be above one [63], around unity [67], or less than one [4]. Roberts [60] argued that possible reasons for different estimates of income elasticity of health expenditure include: model specifications (e.g., cross-sectional vs panel; and static vs dynamic); variable selections; and treatments for the unobserved heterogeneity across countries. Getzen [32] argued that the income elasticity of health care has the characteristics of a necessity goods at the individual and household levels and that of a luxury goods at national levels. The main reason for a lower income elasticity of health care at the individual level is due to the availability of health insurance. At the same time, insured consumption also then contributes to the rapid growth of health expenditure at the aggregate level [49]. Parkin et al. [54] suggested that purchasing power parity (PPP), instead of exchange rates, should be used to compare health spending and GDP per capita among nations. They also showed that the elasticity estimated varied considerably using different functional forms, some of which produced income elasticity estimates of less than one.

The convergence of health expenditure in developed countries have been examined in only a limited number of previous studies: Hitiris [36]; Barros [6]; Nixon [50]; Hitiris & Nixon [37]; Narayan [44]; Panopoulou & Pantelidis [53]; Lau et al. [41]; Pekkurnaz [55]. In the first study Hitiris [36] argued that convergence in economic development and standard of living among countries can lead to the convergence of health expenditure. However, the author found that health care spending and GDP per capita of European countries in the 1960–1990 period actually diverged (based on significant variations of these variables among countries).

Barros [6] found that cross-sectional dispersions in health spending decrease over time (i.e., evidence of *σ*−convergence) and negative correlation between the growth rate and the initial level of health spending (i.e., evidence of *β*− convergence). However, the characteristics of the health system (e.g., the availability of a gatekeeper, public reimbursement or public integrated system) were found to have no significant effects on either the growth or level of health expenditure. The share of public spending in total health expenditure (negative effects) and the proportion of the population over 65 years of age (positive effects) significantly determined the *level* of health expenditure despite having no significant effect the *growth* of health spending.

Nixon [50] tested for the presence of *σ*−convergence (i.e., less variation in growth rates among countries over time) and *β*−convergence (i.e., countries with lower starting point grow at faster rates) in health expenditure among OECD countries in the 1960–1995 period. The author found that health expenditure of OECD countries indeed converged in the study period for both *β*− and *σ*− convergence measures. Similar findings were also obtained by Hitiris & Nixon [37] using data of EU member countries for the period of 1980–1995.

Narayan [44] applied the Lagrange multiplier unit root test procedure, which allows up to two structure breaks, to explore the stationarity of differences in health spending per capita of the United Kingdom, Canada, Japan, Switzerland, and Spain with the USA over the period 1960–2000. The author found significant evidence that health expenditures in the six countries converged. However, he did not find evidence of convergence when applying standard unit root tests with no structural breaks.

Panopoulou & Pantelidis [53] was the first study that applied the Phillips & Sul [58]’s approach to examine the convergence of health expenditure of 19 OECD countries in the period of 1972–2006. They found no evidence of overall convergence, which was mainly due to the faster growth of health expenditure in the USA. The authors also conducted a convergence test for five components of the health expenditure per capita: health expenditure per GDP, labour productivity, employment rate, activity rate, and the proportion of working age population. They found full convergence in employment rate, activity rate and the proportion of working age population but divergence in the proportion of health expenditure in GDP and labour productivity. The authors also applied the test on health outcomes, macroeconomic, demographic, and lifestyle indicators. Their results reveal that overall convergence only arose for selected factors, including the infant mortality rate (health outcomes), GDP per capita, inflation (macroeconomic indicators), the dependency ratio, the labour participation rate for females (demographic indicators), and alcohol consumption (lifestyle indicators). Although testing for the convergence of health expenditure determinants empirically has been useful investigations, we believe that theoretical foundations are required to link the convergence tests in these factors.

Lau et al. [41] applied a non-linear panel unit root test to examine the convergence of health expenditure among 14 EU countries during the 1970–2008 period. The authors did not find significant evidence that health expenditure in the selected EU countries converge, which is in contrast to the simpler *β*− and *σ*− convergence tests by Nixon [50] and Hitiris & Nixon [37]. Pekkurnaz [55] also applied a non-linear panel unit root test to investigate the convergence of health expenditure of 22 OECD countries in the 1980–2012 period. Similar to Lau et al. [41], the author could not reject the null hypothesis of a unit root in health expenditure, indicating no overall convergence. The findings from these studies highlight the importance of taking into account the non-linearity and dynamics in health expenditure.

In summary, there were only a limited number of previous studies that examined the convergence of health expenditures among developed countries. The test procedure in most previous studies included *β*− and *σ*− convergence which were based on restrictive assumptions that countries follow the same growth path due to, for example, having common technology, similar preference, policies and potential for growth. The unit root test approach applied by Narayan [44] was more flexible but it only allows up to two structural breaks in the form of dummy variables (i.e., level breaks). More recent studies include the dynamic convergence test by Panopoulou & Pantelidis [53] and non-linear panel unit root tests by Lau et al. [41] and Pekkurnaz [55] but they did not follow-up with analysis on determinants of health expenditure growth. This study contributes to the literature by applying a dynamic economic growth model proposed by Phillips & Sul [58] followed by panel analysis on factors determining the growth of health expenditure in OECD countries.

## Methods

### Economic growth and health care expenditure

Despite the extensive body of literature on health expenditure growth^1^, very few studies have specified a specific theoretical model to test [60, 72]. This study discusses the inter-connectedness between health expenditure and economic development and applies a dynamic economic growth model to examine the sources of growth in health spending, and the (null) hypothesis of (no) growth convergence among developed countries.

Health care plays an important role in economic development because it may help to ensure a healthy and productive labor force for the economy [34]. It is obvious that when workers have good health, they are less likely to be absent from work due to sickness, and hence, become more productive at producing goods and services, *cereris paribus*.^2^ Microeconomic theory (see, for example, Baumol & Blinder [8]) suggests that increased income raise the demand for health services (via income and substitution effects), especially in respect of elective services such as cosmetic surgery. This behavior can also be explained by the health capital concept proposed by Grossman [33], which suggests that individuals tend to invest for further health improvement when income increases such that their improved health stock would be available to generate more wealth in the future. Ironically, income increases may also lead to further increases in health care consumption due to the emergence of “diseases of affluence” such as obesity, strokes and cancer (see, for example, Van de Poel et al. [69]). Economic development may lead to an aging population because life expectancy increases [42] and fertility declines due to, for example, increases in the direct and opportunity costs of having children [12, 25, 64]. Since people often incur high health expenditure at the end of their lifetimes, an aging population is one of the factors contributing to the rising health spending, especially in developed countries [40]. Health expenditure is also affected by lifestyle factors in affluent societies such as the over-consumption of high-energy food, and the lack of physical activity. However, the main factor that drives both economic development and health expenditure is technological progress because, for example, new technologies offer firms, including health services providers, an opportunity to earn monopoly profits [26, 52, 65].

Based on economic growth models (e.g., Solow [66]), the production of health services can be represented as a function of labor (e.g., doctors, nurses and allied health workers) and capital (e.g., buildings, beds and medical equipment). In the early stage of economic development, labor and capital are the main contributors to the amount of health care services provided. Based on this concept, indicators such as ratios of doctors and number of hospital beds per 1000 population are still used to measure the development of health services. Endogenous growth models (e.g., Nelson & Phelps [46]; Romer [61, 62]), however, suggested that technological progress is more important to economic growth, especially in the long-term. In the health sector, technological progress allows the treatment of new diseases or makes current treatments more effective, and hence more health care services are produced. However, the process of inventing new technologies often involves more time and resources than the process of learning from existing technologies, and hence health expenditure growth rates of countries can converge over time as developing countries adopt new technological advances in their production process. Other factors that drive the convergence of health expenditure growth are the diminishing returns to capital and labor: the amount of output produced increase at decreasing rates as labor and capital increases [5, 10, 11, 23].

The economic growth literature refers mainly to two types of convergence: *β*− convergence and *σ*− convergence which, together, explain how developing economies can “catch up” (e.g., by adopting existing technologies) with developed economies. The *β*−convergence refers to a negative correlation between the initial level of real income per capita (a proxy for economic development) and its growth over time, which occurs when economic growth rates of developed countries tend to be slower than that of developing countries. The *σ*−convergence refers to the reduced dispersion of growth across countries over time (as measured by the coefficient of variation). Another approach to examine the convergence of growth is testing for the stationarity of the differences in growth rates between countries. If the difference in the growth rate of two countries is stationary, the pair converges. In this study, we follow the economic growth model by Phillips & Sul [58] which allows heterogeneity with different transition paths among countries and also enables one to identify convergence among sub-groups of countries.

### The *logt* convergence test

Based on the dynamic growth model developed by Phillips & Sul [58] we argued that the growth rates of health expenditure of a country are determined by accessibility to common technology (which is available to all countries) and the individualised factors of the country (e.g., the ability to conduct research and development to extend technological progress in the health care sector). This argument can be represented as:

1 $$ y_{it}=\delta_{it}\mu_{t}+\varepsilon_{it}  $$

where *y*_*it*_ is a measure of growth in health expenditure for country *i* at time period *t*, *μ*_*t*_ is the growth contributed by the common technology, *δ*_*it*_ is the individual growth factor of country *i*, and *ε*_*it*_ represent random shocks. The health expenditure growth of countries converge when the individual growth factor (*δ*_*it*_) converges. Assume that the cross-sectional average growth rate of all countries at any period represents the common growth factor (*μ*_*t*_), we can isolate *δ*_*it*_ by taking the ratio of a growth rate of a country and the average rate:

2 $$ h_{it}=\frac{y_{it}}{N^{-1}\sum_{i=1}^{N}y_{it}}=\frac{\delta_{it}}{N^{-1}\sum_{i=1}^{N}\delta_{it}}  $$

The coefficient *h*_*it*_, which was referred to by Phillips and Sul as the ‘relative transition path’, measures the performance of country *i* relative to the growth rate achieved by using the common technology *μ*_*t*_. In this formulation, the overall convergence of all countries is achieved when *h*_*it*_→1 for all *i* as *t*→*∞*. In particular, this condition states that convergence is achieved at the long run (*t*→*∞*) when the difference between common growth factors and individualised growth factors is minimal. This convergence condition can also be expressed as the mean squared of relative transition differences:

3 $$ H_{t}=N^{-1}\sum\limits_{i=1}^{N}(h_{it}-1)^{2}  $$

In this representation, the growth rate of countries converges if *H*_*t*_→0 as *t*→*∞*. When *H*_*t*_ remains positive as *t*→*∞* it is possible for the growth rates of all countries diverge or some countries converge despite divergence occur among all countries. To formulate the test for the hypothesis of convergence, Phillips and Sul [58] employed a semiparametric model for the transition coefficients that allows for heterogeneity over time and across countries as:

4 $$ \delta_{it}=\delta_{i}+\sigma_{i}\xi_{it}L(t)^{-1}t^{-\alpha}  $$

where *δ*_*i*_ is a time-invariant growth factor for country *i*, *ξ*_*it*_ is identically and independently distributed with mean of zero and variance of one across *i*, but weakly dependent over *t*, and *L*(*t*)^−1^ is a slow decay function such as the logarithm of *t* for which *L*(*t*)^−1^→0 as *t*→*∞*; *σ*_*i*_ is an idiosyncratic scale parameter; and *α*≥0 is the decay rate. Equation (4) suggests that the condition for convergence is a slow decay component in the growth rate trajectories of individual countries. Under this specification, the null (convergence) and the alternative (non-convergence) hypotheses are expressed as:

5 $$ \left\{\begin{array}{ll} H_{0}:\delta_{it}=\delta & \text{for all } i\\ H_{A}:\delta_{it}\ne\delta & \text{for some } i \end{array}\right.  $$

The alternative hypothesis can also be specified to test for the formation of sub-convergence groups. For example, the alternative hypothesis for the formation of two sub-convergence groups is specified as:

6 $$ H_{A}:\delta_{it}\rightarrow \left\{\begin{array}{ll} \delta_{1} & \text{if } i\in G_{1}\\ \delta_{2} & \text{if } i\in G_{2} \end{array}\right.  $$

where $\delta _{1}={\lim }_{N\rightarrow \infty }N_{1}^{-1}\sum _{i\in G_{1}}\delta _{it}$ and $\delta _{2}={\lim }_{N\rightarrow \infty }N_{2}^{-1}\sum _{i\in G_{2}}\delta _{it}$, *N*_1_ and *N*_2_ are the number of countries in Group 1 and Group 2 such that *N*_1_+*N*_2_=*N*, which is the total number of countries.

Using the limiting form for the quadratic difference $H_{t}\sim \frac {A}{L(t)^{2}t^{2\alpha }}$ as *t*→*∞* for a constant *A*>0 and setting the decay function *L*(*t*)=*log*(*t*), Phillips and Sul [58] proposed the test for convergence in the form of a regression of ‘log *t*’ as:

7 $$ log\frac{H_{1}}{H_{t}}-2log(log\,t)=\omega+\gamma log\,t+u_{t}  $$

where *u*_*t*_ is the random error. The null hypothesis of convergence is rejected (i.e., health expenditure growth rates of countries diverge or only converge among sub-groups) if *γ* is less than zero, while a non-negative *γ* suggests that convergence occurs among all countries. Thus, the test for convergence is now simply a heteroscedasticity and autocorrelation consistent (HAC) one-sided *t*-test for the null hypothesis that *γ*≥0 by estimating Eq. (7) with Newey & West [47] robust standard errors. In this study, we choose the five percent level of significance for the test, hence, the null hypothesis of convergence is rejected if *t*-value of parameter *γ* in Eq. (7) is less than or equal to –1.65. When the null hypothesis of overall convergence is rejected (i.e., *γ* is negative and significant), one can then test for the formation of sub-convergence groups using a four-step clustering algorithm by Phillips and Sul [58] as follows:

1. Order countries in the sample according to the health expenditure growth in the last period.
2. Form a core group of *k* ^∗^ countries by selecting the *k* countries with the highest health expenditure growth rate to form a sub-group *G*_*k*_ and run a convergence test. The optimal size of sub-groups is determined by maximizing the *t*-value of the *γ* coefficient using the *k* countries (*t*_*k*_) such that *k* ^∗^=*arg* *max*_*k*_{*t*_*k*_} subject to *min*{*t*_*k*_}>−1.65.
3. Add one country at a time to the core group and run the convergence test, the country is added if the *t*-value concludes that *γ* is non-negative.
4. Repeat the process in steps 1 to 3 for the remaining countries. If there is no *k* in step 2 that satisfies *t*_*k*_>−1.65 then the remaining countries do not form any sub-convergence group.

Applying the above procedure we are able to test whether health expenditure of selected OECD countries in 1975–2004 converge, diverge totally or form convergence groups. To examine factors that may affect the trends and patterns of growth in health expenditures we apply panel data analysis.

### Determinants of health expenditure

To examine the determinants of health expenditure, we propose a panel data specification as follows:

8 $$ Hexp_{it}=\beta_{0}+\beta_{1}GDPcap_{it}+\gamma X_{it}+\delta trend+\alpha_{i}+\epsilon_{it}  $$

where *Hexp*_*it*_ is the logarithm of real health expenditure per capita of country *i* in period *t*; *GDPcap* is the log of real GDP per capita (i.e., GDP per capita in 2000 prices); *X*_*it*_ is the set of other covariates (e.g., proportion of people over 65 years old, share of public expenditure in total health care spending) which are also expressed in natural logarithms; *α*_*i*_ are country-specific fixed-effects; *trend* is the time trend representing technological progress in the study period; and *ε*_*it*_ are random errors. In order to obtain reliable estimates of Eq. 8, we apply panel data methods (e.g., random and fixed-effects estimators) to remove the effects of country unobserved characteristics (*α*_*i*_). Because health expenditure and GDP per capita may be affected by the same source of external shocks, it is possible that cross-sectional correlations exist in the data set. We examine this issue by applying the cross-sectional dependence test by Pesaran [56]. It is also possible that the error term (*ε*_*it*_) is serially correlated and has non-constant variance, thus we apply the test for the serial autocorrelation by Wooldridge [74] and the likelihood ratio test for heteroscedasticity. Finally, we apply the Hausman specification test to select between the random effects and fixed effects estimators of Eq. 8.

## Data and variable selections

Most variables for this study were selected from the OECD health data 2007 [18]. To form a balanced panel data for the analysis, we chose 21 countries and the period 1975–2004 based on the availability of data on health total expenditure per capita (a lot of missing data pre-1974 and in 2005, based on the 2007 version of OECD health data set) and lifestyle variables such as calories intake, fat intake and alcohol consumption (available only up to 2004). We also use data from the Penn World Table version 7.1 [35] to explore effects of macroeconomic indicators on the growth of health expenditure.

We selected health expenditure per capita measured as $US in purchasing power parity (PPP) at 2000 prices to avoid the effect of exchange rates and inflation. Similarly, we selected the real GDP per capita ($US PPP 2000 prices) to examine the relationship between health expenditure and income. It is expected that GDP per capita has a positive association with health expenditure per capita due to both the income and substitution effects discussed earlier. The proportion of people over 65 years of age is selected to represent the effects of population aging on health expenditure, which is also expected to be positive. The data set also contains information on life expectancy, but this variable is highly correlated with real GDP per capita, so we chose not to include it in our estimates. The unemployment rate is also included to capture the possible effects of economic activities on health. We expect higher rates of unemployment to be negatively associated with health due to, for example, stress and lower access to market inputs; and hence, the we expect the association between the unemployment rate and health expenditure to be positive. The consumption of food, as measured by calorie intake per capita, is another input at our disposal Since we believe that, in the OECD countries food shortage is by-and-large not a major problem, calories intake in this group of countries is expected to be positively associated with health expenditure, due to lifestyle-related health problems associated with obesity, for example. In this respect, the data set also contains information on the amount of sugar and fat consumption. We did not include these variables in the analyses because they are highly correlated with calorie intake. Finally, the dataset also indicates the public sector share of health expenditure. We include this variable as an indicator of the extent to which health sectors are “nationalised” or subsidised publicly. Such measures may be important for controlling health expenditure growth, as many countries that provide extensive public subvention of health care (e.g., Australia, New Zealand, Canada and the UK) also use centralised decision-making to determine which services and pharmaceuticals are eligible for subsidies. Furthermore, in some of these systems, the monopsony power of governments operating in the health sector may also serve as a brake on health expenditure growth.

**Fig. 1:**
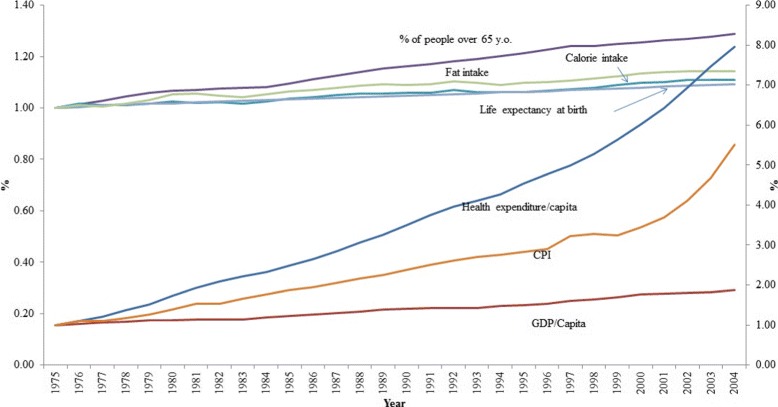
Commutative growth rates of selected variables (secondary verticle axis is used for : health expenditure per capita, CPI and GDP per capita)

The data show that health expenditure per capita experienced the highest growth in the study period, about double the growth of the consumer price index (CPI) and more than four times the growth of GDP per capita in the study period (see Fig. 1, right axis). For example, on average, health expenditure per capita in OECD countries in 2004 was 8 times larger than that in 1975. The relative figures for CPI and GDP per capita growth are five- and two- fold, respectively. The growth rate of other selected indicators are substantially lower: the proportion of people over 65 years old increased by 30% between 1975 and 2004, while the increase of life expectancy was 10 per cent (see Fig. 1, left axis). More importantly, the growth of health expenditure growth rate and CPI seems accelerate rapidly since 2000, while the growth of GDP per capita remained stable. In the analysis, we use CPI and exchange rate to adjust for monetary figure over time and across countries.

## Results and discussion

### Convergence

The “log *t*” test revealed that there is no significant evidence of overall convergence of real health expenditure per capita among OECD countries in the 1975–2004 period (i.e., *γ* is negative and significant) as the *t*-value of “log *t*” is –64.3 (see Table 1). One possible reason for no overall convergence is due to the substantial level of heterogeneity among OECD countries in both economic development and health expenditure. However, we found that there are three groups within which health spending of countries converge (i.e., *γ*≥0): the estimation of *γ* parameter is positive for Group 2 and not significantly different from zero for the remaining groups. The average annual growth rate of real health expenditure per capita is highest among countries in Group 1 (7.9%) and lowest in Group 3 (5.8%). With the exception of Luxembourg, Norway and the USA, countries in Group 1 are relatively poorer OECD countries. The presence of Norway and the USA in Group 1 is consistent with the finding by Panopoulou & Pantelidis [53] but we differ slightly in the ranking other countries, which could be due to the small differences in the number of countries and time period. Note that the convergence test conducted here is a univariate analysis. Thus, other factors such as ageing population, availability of new but expensive treatment options for complicated diseases such as viral hepatitis, HIV and tuberculosis are not taken into account.

**Table 1:** The *log* *t* convergence test

Group	Countries	“log *t*” test		Average annual growth rate
		Coef. (*γ*)	t-stat	
1	Luxembourg, Norway, Portugal, Turkey, Iceland, Ireland, Spain, United States	-0.072	-1.010	***0.079
2	Austria, Belgium, Japan, United Kingdom	***1.969	8.477	***0.069
3	Australia, Canada, Finland, Netherlands, Switzerland, Denmark, Germany, New Zealand, Sweden	0.031	0.224	***0.058
	All countries	***-1.846	-64.332	***0.068

Note: ***, **, and * refers to 1%, 5% and 10% significant level, respectively

**Fig. 2:**
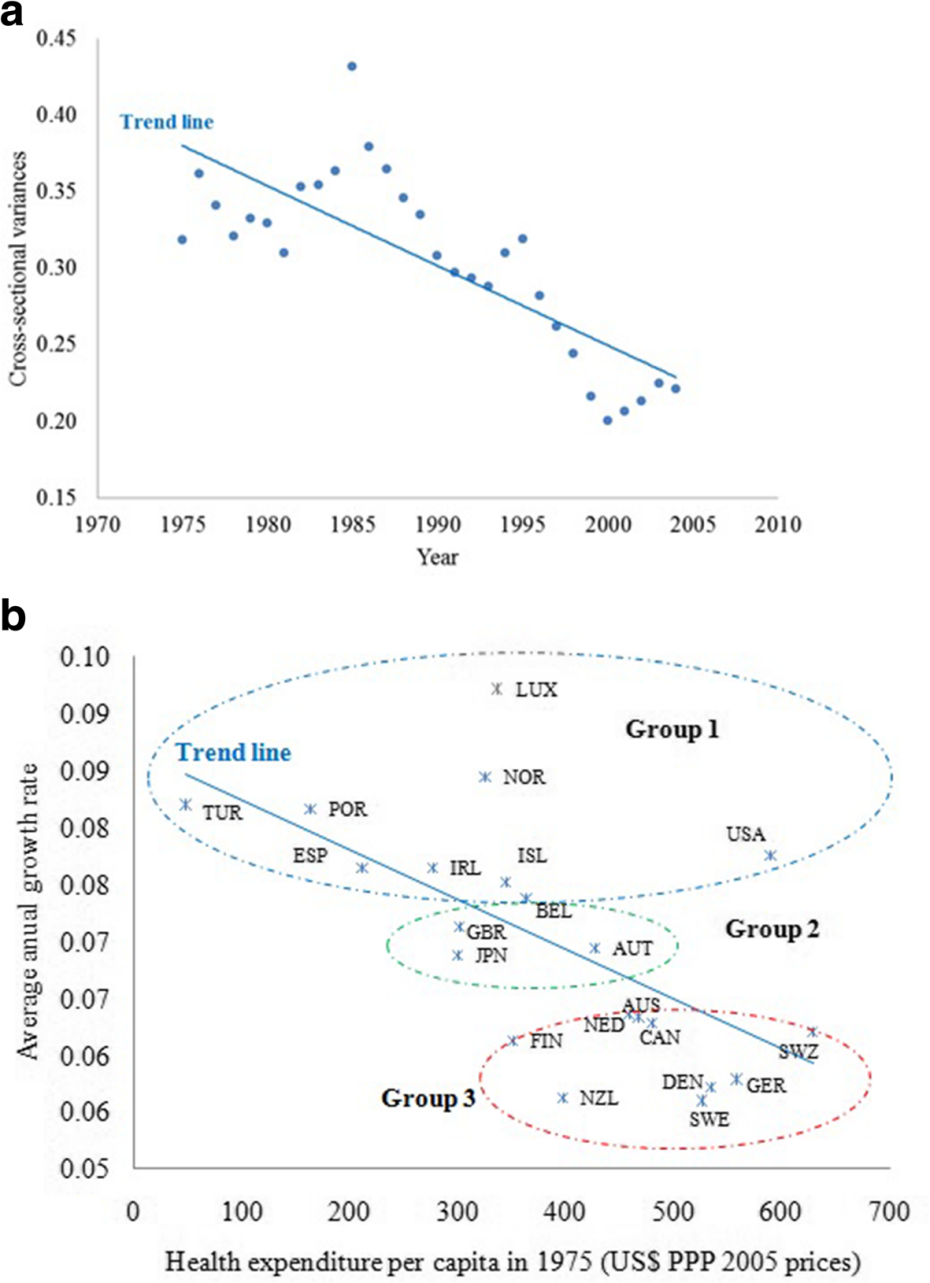
Convergence tests. **a** *σ*-convergence (variations among countries decline over time), **b** *β*−convergence (poorer countries have higher growth rates)

For comparison with previous studies, we also conducted *β*− and *σ*− convergence tests. The results of all these tests show significant evidence of overall convergence. For example, cross-sectional dispersion in health expenditure of OECD countries reduce significantly over time, suggesting that *σ*−convergence is present (Fig. 2 a). We also found a significant negative correlation between the average growth rate of health expenditure and the level of expenditure in the starting year of 1975, suggesting that *β*−convergence exists (Fig. 2 b). A comparison of the results of the *β*−convergence test with that of the *log* *t* test in Fig. 2 b confirms that countries in Group 1, with the exception of the USA, had the lowest health expenditure per capita in 1975, but the highest average growth rate. By contrast, the countries in Group 3 had highest starting health expenditure per capita in 1975, but the lowest average growth rate thereafter.

### Determinants of health expenditure

We apply standard panel data econometric methods to examine the determinants of health expenditure. To explore the possible differences among convergence groups, we conducted the analysis separately for each group. An application of the Im et al. [38] test for unit roots rejected the null hypothesis that the panel contains a unit root (see Table 2). However, the test for serial correlation and cross-sectional dependent rejected the null hypothesis of no serial correlation and cross-sectional independent, respectively. Finally, the Hausman specification test rejected the null hypothesis that parameters of the random- and fixed-effects estimators are the same, suggesting that a fixed-effects estimator, which takes into account the effects of unobserved country characteristics, is preferred.

**Table 2:** Factors determining health expenditures in OECD countries

Variables	Fixed-effects		Random-effects	
	Coef.	Std.err	Coef.	Std.err
Log of real GDP/capita	***0.900	0.067	***1.016	0.057
Log of elderly ratio	**0.167	0.068	***0.237	0.062
Log of calories consumption	***0.800	0.154	***0.820	0.151
Log of unemployment rate	***0.092	0.013	***0.100	0.012
Log of public share of total health expenditure	***0.339	0.049	***0.308	0.048
Trend line	***0.042	0.002	***0.039	0.002
Constant	***-11.215	1.520	***-12.549	1.433
	*Unit root test*	*Auto-* *correlation test*	*Cross-sectional independent*	*Hausman test*
Test statistics	254.6	65.5	23.2	151.1
*p*-value	0.00	0.00	0.00	0.00

Note: Significant levels are ***=1%, **=5% and *=10%

The regression results show that the income elasticity of health care expenditure is less than one. The random-effects estimator, however, produce an income elasticity of slightly greater than one. This result suggests that, ignoring unobserved country characteristics may over-estimate the income elasticity of health expenditure and conclude wrongly that health care is a luxury good. Thus, our results suggest that health care is a necessity, not a luxury, at the national level: a one percent increase in GDP per capita is associated with 0.9% increase in health expenditure per capita.

The results also show that the most significant parameter is the trend line, a proxy for technological progress over time. This result is consistent with the literature to date. In particular, parameters of the trend line show that the average growth rate of health expenditure per capita in the 1975–2004 period is four percent per year. All remaining covariates are also significantly contribute to the increase of health expenditure. In particular, calorie intake is the most substantial determinant of health expenditure with the elasticity of 0.8. The most likely reason for this association may be due to the costs of obesity-related health problems. Public share of health expenditure also play an important role in the total health expenditure with the elasticity of 0.33, which is as expected for developed countries in the sample. Developed countries also face aging population, which may contribute to the rising costs of health care. In particular, an increase of the elderly ratio by one percent is associated with 0.17% increase in health expenditure. Unemployment is also significantly associated with health expenditure but the magnitude of elasticity is minimal, at 0.09.

## Conclusions

This study has examined the trend of health care expenditure for OECD countries during the period 1975–2004. Adapting a dynamic economic growth model developed by Phillips and Sul [58], we tested the hypothesis that the growth of health spending per capita in these countries converge over time. We did not find significant evidence of overall convergence in the health spending growth among the countries for the study period, but identified three sub-groups of countries which tended to converge over this period. Using a fixed-effects estimator, we find that the rate of growth in health care expenditure per capita is less than that of GDP per capita (i.e., health care is a necessary goods). The main driver for increasing health expenditure is technological progress, which accounts for four percent per year and accelerated faster after each decade in the study period. This result is consistent with the existing literature on health expenditure growth. The results of our paper therefore suggest that the explanations and predictions of health expenditure growth based on existing models is unlikely to be affected by convergence. This result is important, because it suggests that policy-makers in lower health expenditure countries need not be concerned about convergence *per se*frustrating attempts to contain health expenditures. Yet it also means that policy-makers in high health-expenditure countries should not depend on convergence to help contain health expenditure growth at home. Rather, microeconomic initiatives that target the modifiable sources of health expenditure growth–particularly health technology diffusion and insurance–are likely the only solutions to containing the growth of health expenditure in high-income nations.

## Endnotes

^1^ See, for example, Gerdtham & Jonsson [28] for a comprehensive review of these studies.

^2^ The *ceteris paribus*condition assumes that other factors (e.g., proportion of elderly people and their higher share of health expenditure) among OECD countries remain constant.
